# Comparison of preemptive ibuprofen, acetaminophen, and placebo administration in reducing peri‐ and postoperative pain in primary tooth extraction: A randomized clinical trial

**DOI:** 10.1002/cre2.465

**Published:** 2021-06-13

**Authors:** Nabih Raslan, Toufic Zouzou

**Affiliations:** ^1^ Department of Paediatric Dentistry Tishreen University Lattakia Syria

**Keywords:** extraction pain, injection pain, preemptive analgesics, primary tooth extraction

## Abstract

**Background:**

The management of pain resulting from anesthesia injection, tooth extraction and in the period after extraction is of great importance in pediatric dentistry.

**Objective:**

The aim of this study was to compare the efficacy of the preemptive administration of ibuprofen or acetaminophen with placebo in reducing the pain during injection, extraction and postoperatively in children undergoing primary tooth extraction.

**Material and methods:**

A randomized, placebo‐controlled, triple‐blinded clinical trial of cooperative children who needed primary molar extraction by local anesthesia. Sixty‐six children aged between 6 and 8 years were randomly assigned to one of three groups: (a) Acetaminophen syrup (320 mg/10 ml); (b) placebo solution; and (c) ibuprofen syrup (200 mg/10 ml). Each of the three solutions was given 30 min before administration of the local anesthetic agent. The Pain level was assessed using the Wong–Baker faces® pain rating scale after injection, extraction, and postoperatively. The Kruskal–Wallis and Mann–Whitney U test were used to evaluate the pain scores between groups at confidence level of 95%.

**Results:**

The use of preemptive analgesics showed lower pain scores compared to placebo. Additionally, only ibuprofen significantly reduced pain scores compared to placebo at the points immediately after injection (*p* = 0.001), immediately after extraction (*p* = 0.0001) and 5 h after extraction (*p* = 0.002).

**Conclusion:**

Preemptive usage of ibuprofen reduces injection pain and relieves both extraction and postoperative pain in children undergoing primary tooth extraction.

**What this paper or case report adds**

- It adds the knowledge regarding pain relief of injection and extraction in children.
- Preemptive analgesic medications have a beneficial effect on alleviating postoperative pain following tooth extraction in children.
- Ibuprofen is an effective analgesic for postoperative pain relief in children undergoing primary tooth extraction.

**Why this paper or case report is important to pediatric dentists**

- Pediatric dentists may consider preemptive ibuprofen in children before injection and extractions.
- Identifies that Ibuprofen is an effective method of reducing postoperative pain.

## 1 INTRODUCTION

Pain during and after dental procedures is one of the main reasons that children find dental procedures unpleasant. The memory of pain during primary experiences can lead to further dental anxiety and hinder a child's dental treatment in the future (Oliveira et al., 2012). Routine dental treatments in pediatric dentistry have been and are still carried out with ineffective pain control based on the assumption that children do not experience significant amounts of pain (Nakai et al., 2000; Versloot et al., 2004; Wondimu & Dahllöf, 2005).

Ghanei et al. (2018) studied the frequency and reported intensity levels of pain and discomfort in children after dental extraction, it has been shown that 62% of children can experience pain after dental extraction, with injection being the most common given reason for pain. Due to hard and soft tissue damage during the extraction process of the tooth, which results in pain and inflammation (Jürgens et al., 2003; Primosch et al., 1995). Pain and discomfort throughout dental procedures are controlled by anesthesia administration. Unfortunately, the local anesthesia injection itself is considered painful to patients (Ghanei et al., 2018). In addition, the previous studies have reported that the administration of anesthesia results in an insufficient pain reduction during these procedures (Ghanei et al., 2018; Wahl et al., 2001). It is therefore very important to seek ways to reduce pain and discomfort following various dental procedures.

Several studies have been shown that preoperative oral usage of analgesics was beneficial in postoperative pain relief compared to placebo (Gazal & Mackie, 2007; Perrott et al., 2004; Shafie et al., 2018).

Regarding the extraction‐related pain, Dental literature indicates that preoperative administration of analgesics can decrease post‐extraction pain scores in adults (Isiordia‐Espinoza et al., 2012; Pozos‐Guillen et al., 2007). However, a recent systematic review by Ashley et al. (2016) stated that Controversial reports can be observed about the efficacy of pre‐emptive analgesia used on post‐operative pain relief in pediatric populations, and the available evidence is not sufficient to determine whether preoperative analgesics administration can reduce postoperative pain in children after tooth extraction under local anesthetics.

In a study by Primosch et al. (1993), it was found that there was no significant decrease in post‐extraction pain in children between placebo and paracetamol groups. Primosch et al. (1995) also found that preoperative administration of ibuprofen and paracetamol was superior to placebo in pain relief after primary tooth extraction. In a study by Baygin et al. (2011), the preoperative use of ibuprofen and paracetamol showed lower pain scores compared to placebo in children during mandibular primary tooth extraction.

Ibuprofen is one of the most commonly used analgesics, and it exerts anti‐inflammatory, analgesic, antipyretic, and antiplatelet properties in dosages ranging from 10 mg/kg/day to a maximum of 40 mg/kg/day, with an onset of action within 24 to 30 min after administration (Bjørnsson et al., 2003; Mehlisch & Sykes, 2013; Olson et al., 2001; Rainsford, 2009). It was used in adult studies to evaluate its preoperative effect after dental procedures on post‐extraction pain relief (Bjørnsson et al., 2003; Olson et al., 2001). Acetaminophen is also an analgesic with efficacy for mild to moderate pain and is an antipyretic (Becker, 2010). By contrast, acetaminophen is almost completely devoid of anti‐inflammatory activity (Becker, 2010). Several investigations have reported its efficacy in postoperative pain relief after third molar surgery (Bjørnsson et al., 2003; Olson et al., 2001). It was found to be a safe and effective analgesic as an antipyretic agent in dosages ranging from 15–20 mg/kg/day to a maximum of 60 mg/kg/day (Baygin et al., 2011; Sarrell et al., 2006). It is rapidly absorbed from the gastrointestinal tract with an onset of action within 30 min after ingestion (Olson et al., 2001; Sarrell et al., 2006).

The aim of this study was to evaluate the efficacy of premedication with ibuprofen or acetaminophen on the pain of injection, extraction and postoperative pain following primary molars extraction under local anesthesia.

## 2 MATERIALS AND METHODS

The protocol of the study had been approved by the Ethics Committee of the collage of Dentistry Research Centre at the university under approval (2304) during session (15), held on May 22, 2018. It was registered in the clinical trials register of clinical studies with registration number NCT03786029.

### 2.1 Study design

**FIGURE 1:**
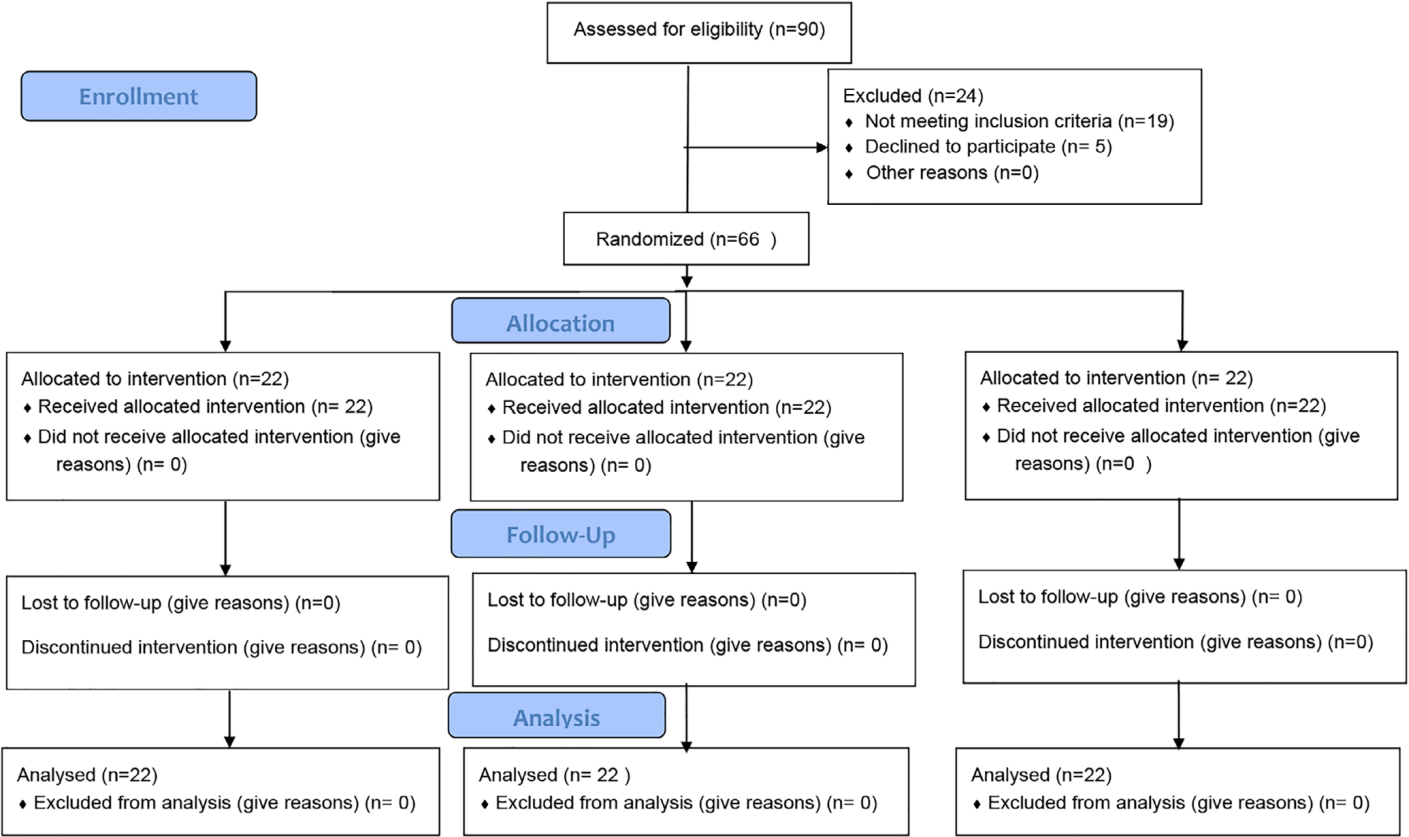
Flow diagram depicting study selection criteria

Sixty‐six children aged 6–8 years old, who needed primary molars extraction, were included in this study (Figure 1). All patients were treated at the Department of Paediatric Dentistry between April 1, 2019, and June 1, 2019. All procedures were approved by the Institutional Review Board (No. 2304). The patients' parents were fully informed about the nature, aim and method of the study and they provided their written consent on behalf of their children to participate in the study. The sample size calculation was based on a previous study in Turkey Baygin et al. (2011) by power analysis using a five‐face scale score (SD: 0.87, effect size 1.149, normal two‐sided test). A sample size of 18 Children per group would be sufficient to get an alpha error of 5% and a power of 95%. Taking into account the probability of dropping out of the sample. Therefore, the sample size was raised to 22 children per group.

The inclusion criteria were as follows: Cooperative child, either positive or definitely positive, according to the Frankl behavioral rating scale, and healthy child (ASA 1) according to the American Society of Anesthesiologists classification (ASA Class) and with no contraindication to receiving either of the two analgesics and/or local anesthesia. The child's age ranges from 6 to 8 years, and weight ranges from 21.5 to 26.5 kg (American Academy of Paediatric Dentistry, 2020). The molars selection criteria included those unfit for restoration or ones with abscess and infection exceeding 1/3 of the inter radicular area.

Exclusion criteria were non‐cooperative children, those with acute pain, patients taking analgesics within 5 h prior to dental extraction and those with a history of prolonged bleeding, hypersensitivity, or allergic reactions to analgesics or any of the drugs tested, patients without a mobile or without parental supervision for the postoperative period. Molars with advanced physiological resorption (more than a third of root length) were also excluded from the study.

Those subjects meeting the selection criteria were given one of the following three solutions: Group 1: Ibuprofen suspension (Ibufen® 100 mg/5 ml; strawberry flavored, red color, Alpha, Pharma); Group 2: Paracetamol Elixir (Paradrin®, 160 mg/5 ml; strawberry flavored, red color, Avenzor, Pharma); Group 3: Strawberry‐flavored placebo solution. The drugs in all of the groups were prepared in a strawberry‐flavored solution of the same color and scent. In order to carry out a triple‐blinded study, the three solutions were placed in bottles identical in shape and were encoded as A (Acetaminophen), B (placebo), and C (Ibuprofen). Only the pharmacist who is not associated with the study and prepared the medications was aware of their contents. The researcher, the child/parent and the assistant were all blind to the content of the bottles.

For randomization, the 66 patients were assigned to the three groups as per a randomized table. In order to conceal the allocation sequence, group identifiers were included in dark and sealed envelopes with session numbers identical to those assigned to patients by the randomization table. The envelopes were kept at the Department of Family and Community Medicine.

**FIGURE 2:**
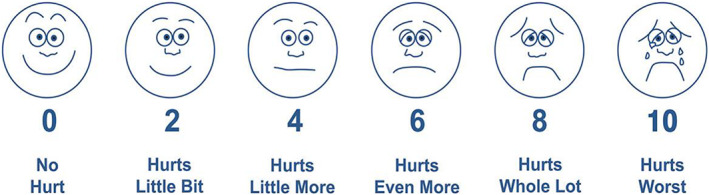
Wong–Baker faces® pain‐rating scale for pain intensity measurement

For the subjective evaluation of pain scores, children were asked to choose the face that best depicts the pain they were experiencing on Wong–Baker faces® pain rating scale (WBFS) (Figure 2). This scale was carefully explained to the children and parents by the researcher in advance.

On session day, the envelope corresponding to a patient number was handed to the assistant. Each patient received an age‐dose volume of the assigned solution in accordance with the American Academy of Paediatric Dentistry's recommendations about the dosing of ibuprofen and acetaminophen based on the age of the child (200 mg ibuprofen/320 mg acetaminophen for children aged 6–8 years) (American Academy of Paediatric Dentistry, 2020). The drug was given by the assistant in the presence of the participant's parents, in identical dosages (10 ml for the three groups) and times – 30 min before administration of the local anesthetic agent. After drug administration, the child was moved to the dental chair, and 1.8 ml of lidocaine 2% with 1:80000 epinephrine (New Static® S.A. Colombia) was injected to obtaining regional adequate anesthesia for one or two molars' extractions. Topical anesthesia was not used for any of the groups. The score was assessed by the child immediately after local anesthesia injection by asking him to choose the face that best depicted the pain that was experiencing on WBFS.

Ten minutes after local anesthesia injection and after ensuring the adequacy of anesthesia (Malamed, 2019), extraction was performed in an uncomplicated manner with minimum surgical trauma. In cases where two adjacent molars needed extraction, it was performed in the same session. After extraction, the score was assessed again. Patients were discharged when considered fit shortly after the 15‐min of extraction. Subsequent pain assessments were evaluated by parents at homely at 1, 2, 3, 4, 5, and 6‐h intervals. Parents were asked to take pictures of the child when he pointed to the corresponding face on the pain scale. Self‐reported pain scores and pictures of the children and the need for analgesics at 1, 2, 3, 4, 5, and 6 h postoperatively points were elicited from the parents by telephone and WhatsApp. Parents were advised to observe their children for lip or cheek biting injuries or bleeding. When such injuries and/or bleeding occurred, the respective child was excluded from the sample.

### 2.2 Data analysis

All recorded pain scores were obtained from the patients included in the study and during all evaluation points. The Chi‐squared test (χ2) was used to analyze the demographic variables, site of extraction and number of the teeth extracted among the groups. Because the observations were independent and the dependent variable “pain score” was ordinal, differences in pain scores between groups were evaluated using the Kruskal–Wallis test. For further pairwise comparison of medication groups, the Mann–Whitney U test in a post hoc manner was implemented. All statistical analyses were performed using SPSS 20.00 (SPSS, Chicago, IL.USA) software. *p* value <0.05 was considered significant.

## 3 RESULTS

Sixty‐six children (37 boys, 29 girls) with mean age of 7.37 ± 0.66 years were involved in this study. In total, 30 maxillary and 49 mandibular primary molars were extracted. There were no significant differences between the groups at baseline with respect to gender, age, site of extraction, and the number of teeth extracted (Table 1).

**TABLE 1:** Demographic variables (*n*), site of extraction and number of the teeth extracted according to groups

Variables	Group A	Group B	Group C	*p* value
Gender (male*/*female)	14/8	13/9	10/12	0.45
Age (year) (mean ± SD)	7,54 ± 0,57	7,27 ± 0,71	7,29 ± 0,70	0.72
One molar extracted	17	17	19	0.68
Two molars extracted	5	5	3	
Upper molars extracted	5	11	8	0.2
Lower molars extracted	17	11	14	
First molars	12	14	14	0.82
Second molars	5	3	5	
First + second molars	5	5	3	

Abbreviation: SD, Standard Deviation.

**FIGURE 3:**
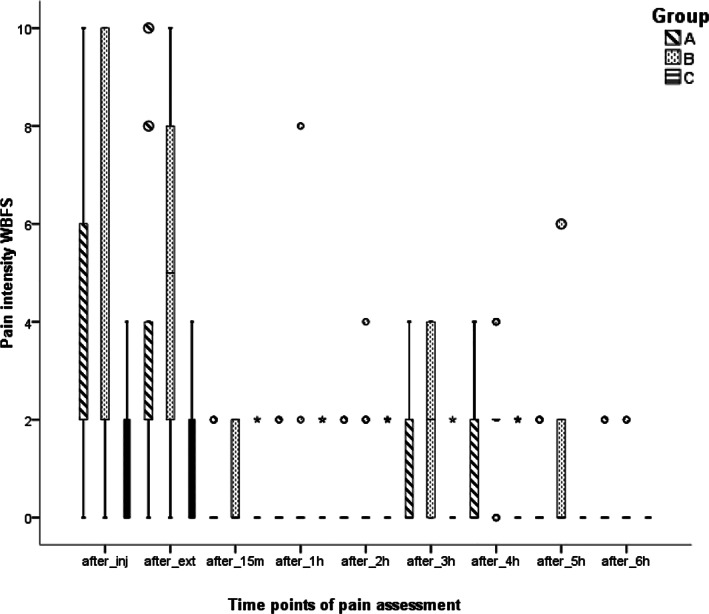
Pain scores (Wong–Baker faces® pain rating scale WBFS) (median values) of the groups in time points

*Pain assessment*. The highest scores of pains were recorded at time points immediately after injection and extraction (Figure 3).

Table 2 shows the median pain scores of the groups including time points. The patients who received preemptive analgesics (Groups A and C) reported significantly less pain than the placebo group (Group B) at time points immediately after injection, immediately after extraction, 3, 4, and 5 h after extraction.

**TABLE 2:** Median postoperative pain score (range) in the three groups

	Group A	Group B	Group C	*p*‐value
After injection	2 (0–10)	2 (0–10)	2 (0–4)	0.001*
After extraction	2 (0–10)	2 (0–10)	2 (0–4)	0.0001*
After 1 h	0 (0–2)	0 (0–8)	0 (0–2)	1
After 2 h	0 (0–2)	0 (0–4)	0 (0–2)	0.4
After 3 h	0 (0–4)	2 (0–4)	0 (0–2)	0.000*
After 4 h	0 (0–4)	2 (0–4)	0 (0–2)	0.000*
After 5 h	0 (0–2)	0 (0–6)	0	0.006*
After 6 h	0 (0–2)	0 (0–2)	0	0.535

* Statistically significant differences.

A comparison of median ranks using the Kruskal–Wallis test showed that the differences in pain scores reduction among the three groups was significant at time points immediately after injection, immediately after extraction, 3, 4, and 5 h after extraction. Post hoc analyses using the Mann–Whitney U test indicated that the pain scores were significantly lower in the ibuprofen group compared to acetaminophen (*p* < 0.05) at the time points previously mentioned (Table 3).

**TABLE 3:** Post hoc analyses between each two groups

	Group A/B	Group A/C	Group B/C
After injection	0.5	0.002*	0.001*
After extraction	0.06	0.001*	0.0001*
After 3 h	0.003*	0.009*	0.0001*
After 4 h	0.007*	0.007*	0.0001*
After 5 h	0.16	0.03*	0.002*

* Statistically significant differences.

Results from chi‐square analysis test showed no significant differences in the median pain scores among the three groups in terms of number and site of teeth extracted (Tables 4 and 5). Additionally, the mean pain scores for males and females were 1.02 ± 0.8 and 1.01 ± 0.9, respectively. There were no significant differences in the mean pain scores among groups in terms of gender (*p*: 0.9).

**TABLE 4:** Median pain score (range) in each group in terms of the number and of teeth extracted

	Group	One molar	Two molars	*p*‐value
After extraction	A	2 (0–10)	4 (0–4)	0.446
	B	4 (0–10)	6 (2–10)	0.189
	C	0 (0–4)	0 (0–2)	0.104
After 3 h	A	0 (0–4)	0 (0–2)	0.940
	B	2 (0–4)	0 (0–4)	0.140
	C	0 (0–2)	–	0.478
After 4 h	A	0 (0–4)	2 (0–4)	0.120
	B	2 (0–4)	2 (0–4)	0.359
	C	0 (0–2)	0 (0–2)	0.238
After 5 h	A	0 (0–2)	0 (0–2)	0.940
	B	0 (0–6)	0 (0–2)	0.493
	C	–	–	0.754

**TABLE 5:** Median pain score (range) in each group in terms of the site of extraction

	Group	Upper molars	Lower molars	*p*‐value
After injection	A	2 (2–8)	2 (0–10)	0.543
	B	10 (0–10)	2 (0–8)	0.016*
	C	0 (0–2)	0 (0–2)	0.973
After extraction	A	4 (0–4)	2 (0–10)	0.446
	B	4 (0–10)	6 (0–10)	0.606
	C	0 (0–4)	0 (0–2)	0.482
After 3 h	A	0 (0–2)	0 (0–4)	0.940
	B	2 (0–4)	4 (0–4)	0.243
	C	–	0 (0–2)	0.815
After 4 h	A	2 (0–4)	0 (0–4)	0.446
	B	2 (0–4)	2 (0–4)	0.243
	C	0 (0–2)	0 (0–2)	0.868
After 5 h	A	0 (0–2)	0 (0–2)	0.940
	B	0 (0–6)	0 (0–2)	0.89
	C	–	–	1.000

* Statistically significant differences.

None of the patients' parents reported any side effects after taking the analgesics or a lip/cheek biting injury in their children.

## 4 DISCUSSION

In this study, preemptive analgesics were compared in relation to their effects in reducing injection and postoperative pain during the first 6 h following primary molar extraction in cooperative children. Cooperative children rated 3 or 4 on Frankl behavior scale were selected to prevent anxiety and fear from overlapping in pain assessment. The selection was also based on how easily the subject was able to follow the dentist's orders and accept undergoing dental procedures, which enabled accurate measurement of pain by self‐assessment throughout the study. For subjective evaluation, the current study used the Wong–Baker faces' pain rating scale. This scale can be easily used in this age group, and it is preferred by children, parents, and clinical practitioners compared with other self‐assessment scales (Raslan & Masri, 2018; Tomlinson et al., 2010).

Previous studies have been conducted on children from a large age range (Baygin et al., 2011; Kharouba et al., 2019; Primosch et al., 1995). Because age characteristics may influence the reports of pain (Gazal & Mackie, 2007; Ghanei et al., 2018; Primosch et al., 1995), this study selected an age range of 6–8 years old, as children in this age bracket have sufficient verbal and cognitive skills to communicate, distinguish and assess pain well by a subjective pain scale like the one used in the study (Wilson, 2013). Moreover, in order to standardize the dose across the three groups in line with AAPD's recommendations on age‐appropriate dosing of ibuprofen and acetaminophen (ibuprofen 200 mg/10 ml and acetaminophen 320 mg/10 ml) (American Academy of Paediatric Dentistry, 2020). Topical anesthesia was not used in any of the groups. Although surface anesthesia may decrease pain of needle insertion (2–3 mm), it is less likely to be effective deeper (Bernardi et al., 1999; Meechan, 2012). According to a study by de Freiras et al. (2015), the topical anesthetic and the placebo had similar effects on pain perception for injection of local anesthesia.

The results of this study showed that only ibuprofen resulted in suppression of injection pain in comparison to placebo. This might be attributed to the fact that ibuprofen is an effective analgesic for children and has potential advantages in pain management compared to acetaminophen (Schachtel & Thoden, 1993). Ibuprofen inhibits sensation of peripheral pain by decreasing PGE2 synthesis, which is locally released during the pain process and is responsible for increasing the sensitivity of nerve endings, whereas acetaminophen is a central analgesic that does not interfere with peripheral prostaglandins synthesis (Dietrich et al., 2015). Moreover, according to a study by Olson et al. (2001), ibuprofen provided significantly faster relief compared to acetaminophen. However, previous studies did not focus on investigating the effect of preemptive analgesics on pain during local anesthesia injection.

Pain results from inflammatory response created by tissue damage. Tooth extraction is the most likely pediatric dental procedure to produce inflammation and pain. In the current study, pretreatment with ibuprofen and acetaminophen exhibited significant differences in pain scores compared to placebo. Moreover, ibuprofen seems to be the most effective analgesic, and it resulted in significantly lower pain scores (*p* < 0.05) compared to that of acetaminophen. Additionally, only ibuprofen significantly reduced pain scores (*p* < 0.05) compared to placebo at the points immediately after extraction and at 5 h after extraction. This is because ibuprofen has excellent analgesic and anti‐inflammatory properties, which are especially important following dental extractions. It acts in the periphery to inhibit the initiation of pain signals by cyclooxygenase inhibition, which in turn prevents prostaglandin synthesis following tissue injury (Dionne et al., 1983). It modulates aspects of inflammation, in which prostaglandins act as mediators by inhibiting peripheral prostaglandin synthesis prior to surgical stimulus, which is responsible for postoperative pain (Dietrich et al., 2015; Dionne et al., 1983). Even though acetaminophen is a clinically effective analgesic that inhibits prostaglandin synthesis in CNS, its efficacy on cyclooxygenase in peripheral tissues is less evident, which accounts for its weak anti‐inflammatory activity (Dietrich et al., 2015). This suggests that analgesic drugs that inhibit peripheral prostaglandin synthesis are more effective in suppressing extraction and post‐extraction pain than those that do not interfere with this pathway. These findings were reinforced by the results reported by Baygin et al. (2011), Dionne et al. (1983), and Olson et al. (2001), all of which were obtained by applying preemptive analgesia. These studies found that ibuprofen and paracetamol could significantly decrease pain scores compared to a placebo, and that especially ibuprofen, when given preemptively, reduces the onset and intensity of postoperative pain. Our findings, however, were inconsistent with the results of Primosch et al. (1995), which did not show significant differences in pain scores among the three groups. This may be because in their study the local anesthesia injection was conducted 15 min after analgesics administration, which was insufficient to provide adequate blood levels before the initiation of tissue trauma. In addition, another distinction from ours is that their sample consisted of children aged 2 to 10 (Primosch et al., 1995).

At 1 and 2 h after extraction, there were no statistically significant differences in pain levels among the three groups; we can explain that the effects of local anesthesia had not worn off in most patients at 1 and 2 h after extraction. This finding corresponds with the results of Dionne et al. (1983), which showed minor differences in pain scores among treatments at 2 h after the pre‐medication dose.

Chi‐square analysis showed no differences in pain scores in relation to gender and number of teeth extracted among the groups. The present findings support that gender and the number of teeth extracted had no effect on the results of pain scores. These findings are congruent with those of other studies (Baygin et al., 2011; Primosch et al., 1995).

This study demonstrated that pretreatment with ibuprofen results in suppression of postoperative pain when compared to acetaminophen. Moreover, it adds to the existing knowledge regarding the important effect of ibuprofen in reducing pain of local anesthetic injection, which in turn could raise the child's pain threshold for following dental procedures, such as tooth extraction. In addition, provides a simple, inexpensive and safe strategy for reducing injection and extraction pain in children undergoing primary tooth extraction.

Compared with previous investigations, the current study's sample included narrower age range, which made it possible to standardize the administered dose of analgesics and the local analgesics. Furthermore, it involved maxillary and mandibular tooth extractions, which makes generalizing the findings more likely. In addition, relaying on sending hourly pain self‐assessments directly via the internet enhanced the accuracy of the results. The validation of the findings of the present study is further confirmed by homogeneity of the three groups in terms of age, gender, site of extraction, number of teeth extracted and the level of cooperation of participating children.

## 5 CONCLUSION

Based upon the results of this study, the following conclusions were drawn:

1. The present study showed that preemptive analgesic administration may be considered a routine and rational pain management strategy in primary tooth extraction procedures in children.
2. Ibuprofen is more effective than acetaminophen in reducing children's pain following extraction of teeth under local anesthesia.
3. Ibuprofen pretreatment suppresses the intensity of injection pain.

## CONFLICT OF INTEREST

All authors declare no conflict of interest.

## AUTHOR CONTRIBUTIONS

We declare that all authors have made substantial contributions. Dr. Nabih Raslan designed the research. Dr. Toufic Zouzou performed the research including extraction teeth and data collection. Dr. Nabih Raslan and Dr. Toufic Zouzou analyzed the data. Dr. Toufic Zouzou prepared the manuscript draft with important intellectual input from Dr. Nabih Raslan. Dr. Nabih Raslan revised the manuscript. All authors approved the final manuscript.

## Data Availability

Data available on request due to privacy/ethical restrictions The data that support the findings of this study are available on request from the corresponding author. The data are not publicly available due to privacy or ethical restrictions.
